# Metabolic activities affect femur and lumbar vertebrae remodeling, and anti-resorptive risedronate disturbs femoral cortical bone remodeling

**DOI:** 10.1038/s12276-020-00548-w

**Published:** 2021-01-12

**Authors:** Mi Yeong Kim, Kyunghee Lee, Hong-In Shin, Kyung-Jae Lee, Daewon Jeong

**Affiliations:** 1 Laboratory of Bone Metabolism and Control, Department of Microbiology, Yeungnam University College of Medicine, Daegu, 42415 Korea; 2 IHBR, Department of Oral Pathology, School of Dentistry, Kyungpook National University, Daegu, 41940 Korea; 3 Department of Orthopaedic Surgery, Keimyung University Dongsan Hospital, Keimyung University School of Medicine, Daegu, 42601 Korea

**Keywords:** Bone, Bone quality and biomechanics

## Abstract

Metabolic activities are closely correlated with bone remodeling and long-term anti-resorptive bisphosphonate treatment frequently causes atypical femoral fractures through unclear mechanisms. To explore whether metabolic alterations affect bone remodeling in femurs and lumbar vertebrae and whether anti-osteoporotic bisphosphonates perturb their reconstruction, we studied three mouse strains with different fat and lean body masses (BALB/c, C57BL6, and C3H mice). These mice displayed variable physical activity, food and drink intake, energy expenditure, and respiratory quotients. Following intraperitoneal calcein injection, double calcein labeling of the femoral diaphysis, as well as serum levels of the bone-formation marker procollagen type-I N-terminal propeptide and the bone-resorption marker C-terminal telopeptide of type-I collagen, revealed increased bone turnover in mice in the following order: C3H > BALB/c ≥ C57BL6 mice. In addition, bone reconstitution in femurs was distinct from that in lumbar vertebrae in both healthy control and estrogen-deficient osteoporotic mice with metabolic perturbation, particularly in terms of femoral trabecular and cortical bone remodeling in CH3 mice. Interestingly, subcutaneous administration of bisphosphonate risedronate to C3H mice with normal femoral bone density led to enlarged femoral cortical bones with a low bone mineral density, resulting in bone fragility; however, this phenomenon was not observed in mice with ovariectomy-induced femoral cortical bone loss. Together, these results suggest that diverse metabolic activities support various forms of bone remodeling and that femur remodeling differs from lumbar vertebra remodeling. Moreover, our findings imply that the adverse effect of bisphosphonate agents on femoral cortical bone remodeling should be considered when prescribing them to osteoporotic patients.

## Osteoporosis: Metabolic links to femur fragility

The metabolic status of post-menopausal women may influence their susceptibility to osteoporosis and their risk of femur fractures when taking anti-osteoporosis drugs. Metabolic activity influences bone density and turnover, particularly following estrogen loss after menopause. Long-term osteoporosis treatment with bisphosphate drugs causes femur fractures in some patients, but the reasons for this are unclear. Kyung-Jae Lee at Keimyung University, Daewon Jeong at Yeungnam University, both in Daegu, South Korea, and co-workers studied three mice strains with phenotypes showing different fat and lean-body masses and found distinct variations between femur and lumbar vertebrae remodeling, with femur remodeling more readily affected by metabolic activity. Both control and estrogen-deficient mice with high levels of body fat exhibited abnormal femur remodeling. Treatment with bisphosphates caused femur fragility in control mice in this group, but not in the estrogen-deficient mice.

## Introduction

Bone remodeling is achieved through a delicate balance between old bone resorption by osteoclasts and new bone formation by osteoblasts^1^. Bone mass and quality progressively decrease during aging because bone destruction exceeds bone formation, resulting in fragility fractures in older adults^2^, and osteoporotic fractures in the geriatric population are emerging as a major health problem^3,4^. In particular, postmenopausal women older than 65 years with estrogen deficiency frequently experience osteoporotic fractures of the hip, pelvis, wrist, humerus, vertebra, and cervical and intertrochanteric femurs^5^. Both older individuals and menopausal women commonly show changes in fatty acid metabolism and body composition (in terms of muscle and fat), with a gradual reduction in bone mass with aging or after menopause and, consequently, a higher risk for bone fractures^6,7^.

Among the various types of bone fractures, atypical femoral fractures (AFFs) are distinct from ordinary femoral diaphyseal and subtrochanteric fractures. According to the revised case definition of the task force of the American Society for Bone and Mineral Research, AFFs are located along the femoral diaphysis (just distal to the lesser trochanter to just proximal to the supracondylar flare) and have several distinctive radiographic features, such as a transverse orientation, minimal or no comminution, and localized periosteal or endosteal thickening of the lateral cortex^8^. AFFs have the clinical characteristics of female predominance and anterolateral femoral bowing. Moreover, ~70% of patients with AFF have prodromal symptoms, such as groin and thigh pain, and 28–62.9% of such patients show bilateral fractures or bilateral radiographic abnormalities^8–10^ and frequently show delayed healing, implant failures, and a need for revision surgery^11,12^. Previous work showed that the implant failure of patients with subtrochanteric AFFs was 23% (7 of 33 cases), and that 33% of patients with AFFs required revision surgery^12^. In addition, in our multicenter retrospective study, we found that the average time to AFF union was 24.9 weeks and that 37% of patients with complete AFFs (17 of 46) failed to show AFF union within 6 months after surgery^11^. These results indicate that bone healing in AFFs is delayed compared to that in typical femoral fractures.

Dual-energy X-ray absorptiometry (DXA) has been widely used to measure bone mineral densities (BMDs) to determine the presence of osteoporosis, to predict fracture risks and to monitor responses to medications. The *T*-score deduced from DXA results is a statistical expression that relates to the number of standard deviations that a patient’s BMD differs from the average value found in sex- and race-matched subjects between the ages of 20 and 30 years, who constitute the reference group. A *T*-score of −2.5 or below for the lumbar spine, total hip, or femoral neck is considered to be osteoporotic, according to the recommendations of the International Society for Clinical Densitometry^13^. In particular, T-score discordance between the spine and hip has been reported in 29–46% of osteoporotic patients^14–18^ and this discordance is relatively increased in patients with AFFs (compared with the general osteoporotic population)^19^. Moreover, this phenomenon is dramatically increased in AFF patients with a history of receiving anti-resorptive drugs such as bisphosphonates^8,20^.

Long-term treatment with anti-resorptive bisphosphonates frequently causes AFFs^8,20^, although the underlying mechanisms have not been fully elucidated. In this study, we investigated the effects of metabolic alterations on bone remodeling in femurs and lumbar vertebrae and whether bisphosphonate risedronate perturbs their reconstruction. We observed differences in trabecular and cortical bone remodeling in femurs versus lumbar vertebrae among the mouse strains and that administering risedronate to mice with a normal bone density resulted in abnormal femoral cortical bones with an enlarged thickness and low bone density; however, these changes were not observed in estrogen-deficient femoral osteoporotic mice. Moreover, our findings suggest that the high frequency of AFFs occurring after bisphosphonate treatment may be due to defective femoral cortical bone remodeling in cases where individuals with normal femoral bone density are subjected to long-term exposure.

## Materials and methods

### Mice

In this study, we used female BALB/cAnHsd, C57BL/6NHsd, and C3H/HeNHsd mice purchased from KOATECH (Pyeongtaek, Korea), which are referred to here as BALB/c, C57BL6, and C3H, respectively. The mice were given ad libitum access to a standard rodent chow diet and normal tap water. The mice were maintained under normal conditions with a 12 h light/dark cycle, a temperature of 20–25 °C, and 60% relative humidity. All animal experiments were approved by the Institutional Animal Care and Use Committees of Yeungnam University College of Medicine and Daegu-Gyeongbuk Medical Innovation Foundation.

### Analysis for metabolic activity

Mice were habituated in metabolic cages (OxyletPro Physiocage System; Panlab Harvard Apparatus, Barcelona, Spain) with free access to water and food, and allowed to acclimate for 24 h, after which various metabolic parameters were measured. Briefly, we monitored activity, food and drink consumption, the volume of carbon dioxide production (VCO_2_), and the volume of oxygen consumption (VO_2_) over a 48 h period. The ambulatory activities of the mice were determined by measuring infrared photocell-beam interruption with the OxyletlPro Physicocage System. VO_2_ and VCO_2_ levels were measured in individual mice at 3 min intervals for 48 h using an O_2_ and CO_2_ analyzer (Oxylet LE 405 gas analyzer, Panlab Harvard Apparatus) at a constant flow rate of 600 ml/min (Oxylet LE 400 air supplier, Panlab Harvard Apparatus). The data were analyzed using Metabolism software, version 3.0 (Panlab Harvard Apparatus) to calculate the respiratory quotient (RQ) as the VCO_2_/VO_2_ ratio and energy expenditure (EE, kcal/[day•kg^0.75^]) as VO_2_ × 1.44 × (3.815 + [1.232 × RQ])^21^.

### High-resolution microcomputed tomography analysis

After the mice were killed, the femurs and fourth lumbar vertebrae (L4) were excised and fixed in 3.7% phosphate-buffered formalin. Subsequently, the bones were transferred to 70% ethanol and dried in air. Trabecular and cortical bone microarchitectures (femoral and lumbar) were assessed by performing high‐resolution microcomputed tomography (µCT) analysis. The µCT images were obtained using a Quantum FX micro CT system (PerkinElmer, Caliper Life Sciences, Hopkinton, MA, USA). The X-ray source was set to a voltage of 90 kVp, a current of 180 μA, and a field-of-view of 10 mm. Each distal femur was scanned in the region proximal to the growth plate and the fourth lumbar vertebra (L4) was scanned in the center of the vertebral body. Three-dimensional structural analyses of trabecular and cortical bone sites were performed using Analyze software, version 12.0 (AnalyzeDirect, Overland Park, KS, USA). Trabecular bone morphometry parameters were calculated for the BMD, bone volume/total volume (BV/TV) ratio, trabecular number (Tb.N), trabecular thickness (Tb.Th), and trabecular separation (Tb.Sp). In addition, cortical bone parameters, including the BMD, cortical area, and cortical thickness, were measured.

### DXA analysis

Body composition and bone mineral analyses were performed with a high-resolution DXA cabinet body composition analyzer (iNSiGHT VET DXA, Osteosys, Korea). The lean body mass was deduced from the total body weight and body fat weight. In color-composition images, fat and lean tissue are indicated in red and green, respectively. In addition, bone mineral contents (BMCs), BMDs, and bone areas were determined in all mice using a cone-beam flat panel DXA detector (iNSiGHT VET DXA, Osteosys).

### Measurement of the mineral apposition rate

For dynamic histomorphometry analysis, calcein labeling was performed to estimate the levels of newly formed bones. Mice were injected intraperitoneally with calcein (15 mg/kg) at 8 and 3 days before killing. Then, the diaphyseal cortical femoral bones were sectioned and calcein double-labeled bone surfaces were photographed to determine the mineral apposition rates (MARs).

### Serum biochemical analysis

To determine the serum levels of bone-related biochemical markers, whole blood samples were incubated at room temperature for 1 h to allow clotting and then centrifuged at 2000 × *g* for 20 min. The resulting supernatants were divided into aliquots and frozen at −80 °C until further analysis. The serum levels of various bone parameters were measured using specific enzyme-linked immunosorbent assay (ELISA) kits according to the manufacturers’ instructions. ELISA kits against the indicated markers were purchased from the following companies: 17-β estradiol and osteoprotegerin from Abcam; osteocalcin and type-I collagen (CTX-1) from Novus Biological; procollagen type-I N-terminal propeptide (P1NP) and parathyroid hormone (PTH) from Cusabio; 1,25-dihydroxyvitamin D_3_ from Cloud-Clone; tartrate-resistant acid phosphatase 5b (TRACP5b) from Immunodiagnostic Systems; and receptor activator of nuclear factor kappa-B ligand (RANKL) from R&D systems. In addition, serum calcium and phosphate levels were determined colorimetrically using a QuantiChrome Calcium Assay Kit (BioAssay Systems, Hayward, CA, USA) and a QuantiChrom Phosphate Assay Kit (BioAssay Systems), respectively, according to the manufacturer’s recommended procedures.

### Ovariectomy

Eight-week-old female BALB/c, C57BL6, and C3H mice were anesthetized by intraperitoneal injection of 2.5% avertin (Sigma-Aldrich, St. Louis, MO) and were subjected to a bilateral ovariectomy to induce bone loss^22^. After an abdominal incision was made around the midpoint between the last rib and iliac crest and after the ovary was removed, the mice were maintained in a warm environment. In addition, the sham group underwent the same surgery, although the ovaries were identified and preserved. Bone indices determined based on µCT images and bone-related parameters in the serum were analyzed at the indicated times. In experiments involving drug administration, 8-week-old female C3H mice were ovariectomized, after which bisphosphonate risedronate (20 μg/kg) was subcutaneously administered to the mice every 2 weeks for 10 weeks. The bone indices were analyzed in the trabecular and cortical bones of femurs and lumbar vertebra.

### Statistical analysis

Quantitative data are expressed as the mean ± SD from the indicated number of mice. Statistical analyses were assessed using Student’s two-tailed *t*-test for comparisons between two groups or analysis of variance and a post hoc test for multiple comparisons using SPSS software, version 21.0. For all experiments, a *p*-value of <0.05 was considered to reflect a statistically significant difference.

## Results

### Variable metabolic activities between BALB/c, C57BL6, and C3H mice

**Fig. 1:**
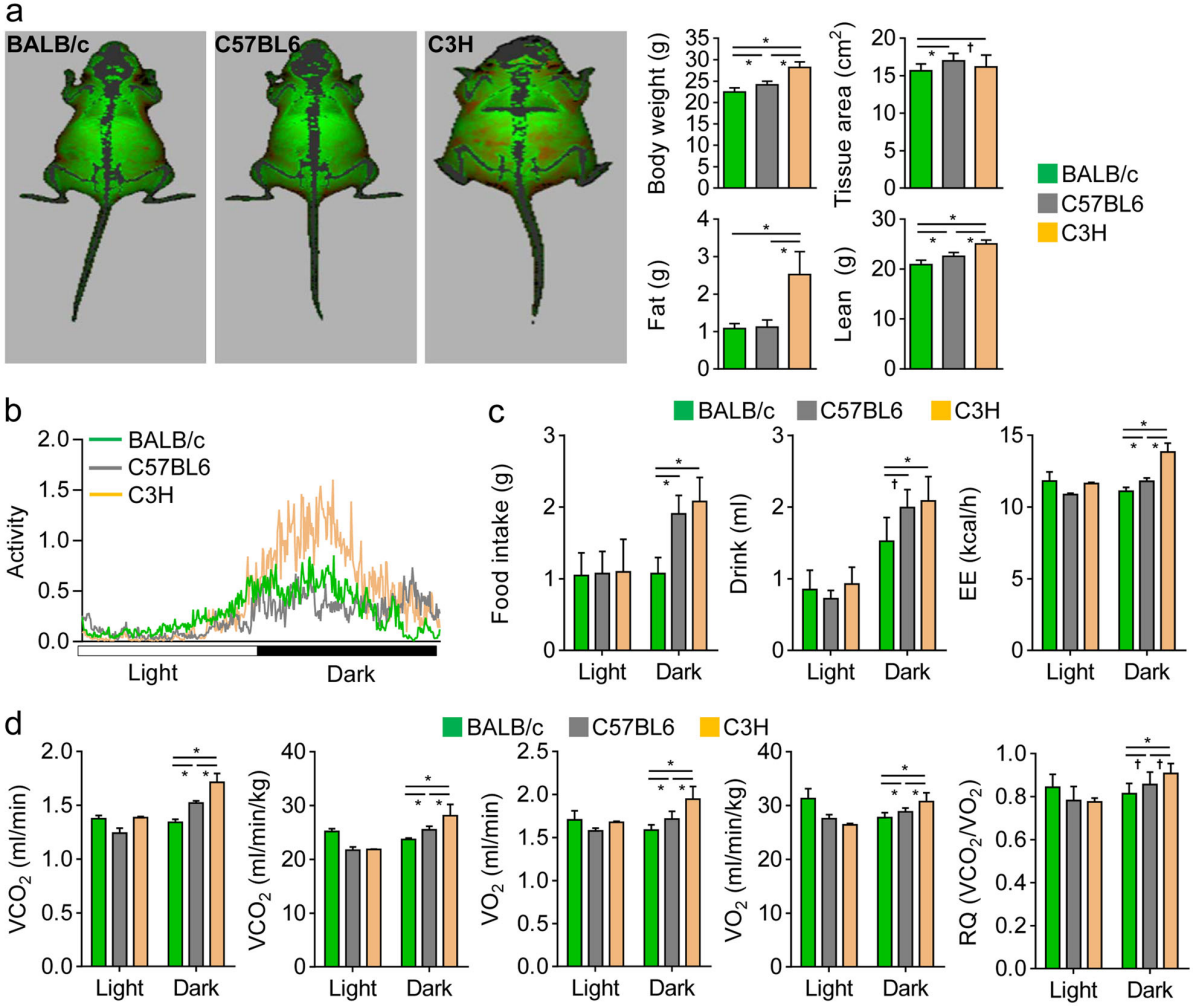
Differences in body composition and metabolic activities among the three mouse strains. **a** Body composition analysis. Representative images and body compositions, including body weight, tissue area, and fat and lean body masses, of 14-week-old female BALB/c, C57BL6, and C3H mice were obtained using a high-resolution DXA analyzer. In the color-composition image, fat tissue is shown in red, and lean tissue is shown in green. **b**–**d** Metabolic activity. Mice were housed in metabolic cages with a resting phase (light) and an active phase (dark), following a standard 12 h : 12 h light/dark schedule. Total activity (**b**), food and drink consumption (**c**), energy expenditure (EE; **c**), carbon dioxide production (VCO_2_; **d**), oxygen consumption (VO_2_; **d**), and the respiratory quotient (RQ; **d**) were monitored for 48 h. The data shown represent the means ± SDs (*n* = 10 mice/group). *P < 0.01; ^†^*P* < 0.05.

Energy metabolism is differentially affected by external and internal factors, such as environmental conditions, personal traits, sex, age, and genetic background^23^. To compare and identify differences in energy metabolism in mice with distinct genetic backgrounds, we studied female mice of three different strains (BALB/c, C57BL6, and C3H). In terms of their metabolic characteristics, it has been reported that BALB/c mice are relatively resistant to high-fat diet-induced obesity and have a low frequency of diabetes due to severe insulin resistance compared to C57BL6 mice^24,25^. In addition, the C57BL6 strain is susceptible to atherosclerosis following atherogenic feeding, but atherosclerosis in the BALB/c and C3H strains is milder than that in the C57BL6 strain^26^. First, we measured the body compositions of mice from all three strains via DXA. As shown in Fig. 1a, the C3H mice exhibited higher body fat than the BALB/c and C57BL6 mice. Moreover, body weights and lean masses were ranked in the following order: C3H > C57BL6 > BALB/c. Second, we analyzed activity, food and drink consumption, and indirect calorimetric values using a metabolic cage to precisely verify metabolic differences between the strains. When the mice were housed under a 12 h : 12 h light/dark cycle to continuously monitor spontaneous activities and identify circadian patterns, their physical activities increased during the dark phase in the following order: C3H > BALB/c > C57BL6 (Fig. 1b). The C57BL6 and C3H mice showed higher food and water consumption and VCO_2_ and VO_2_ levels than BALB/c mice (Fig. 1c, d and Supplementary Fig. S1). Moreover, the RQ (determined as the VCO_2_/VO_2_ ratio) and EE (deduced from the Weir formula using the RQ) were found to increase in the following order: C3H > C57BL6 > BALB/c. Taken together, our results indicate that the BALB/c, C57BL6, and C3H strains have different metabolic phenotypes.

### The bone metabolism of femurs and lumbar vertebrae differed between BALB/c, C57BL6, and C3H mice

**Fig. 2:**
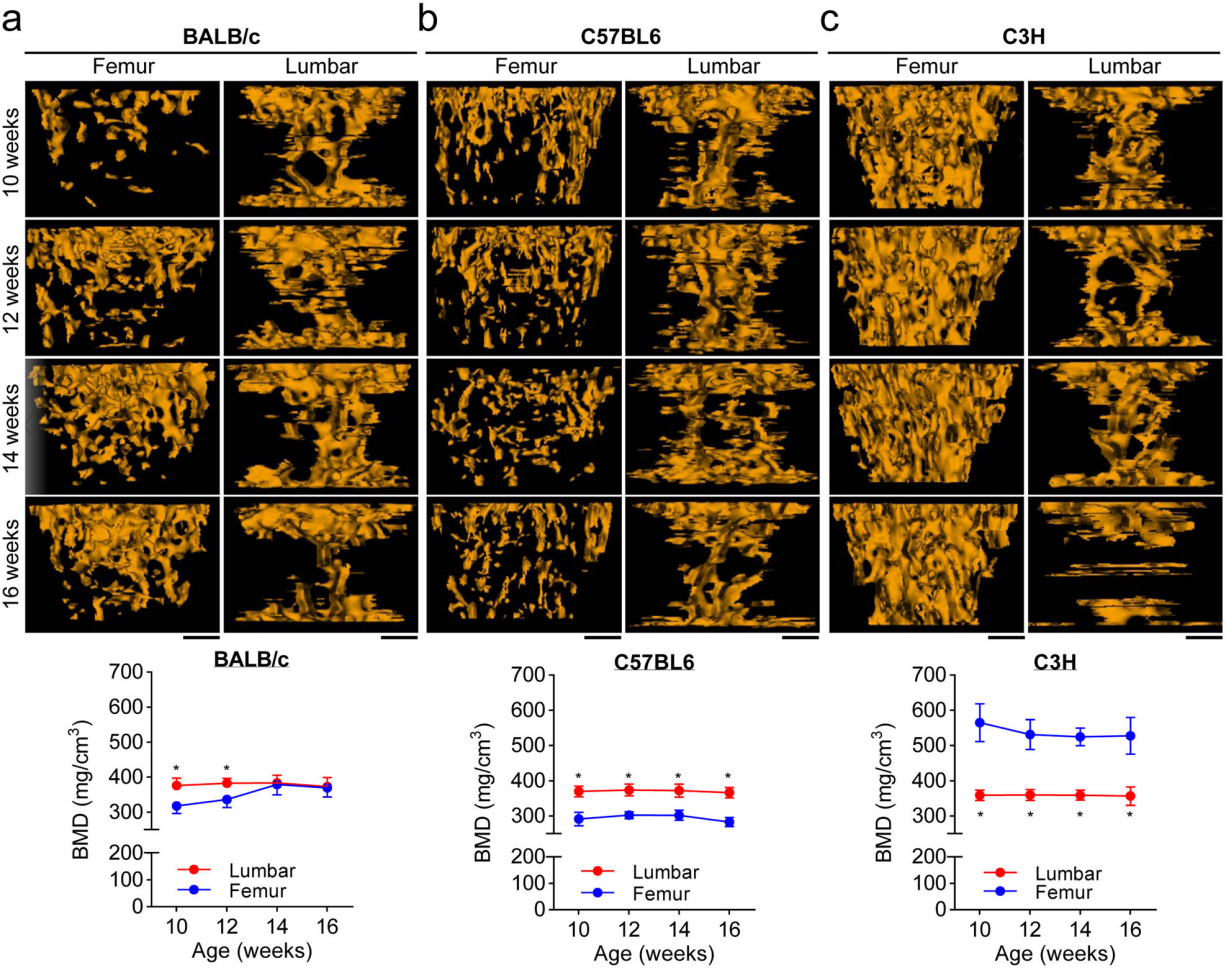
Three-dimensional microcomputed tomography (μCT) analysis of the bone architectures of femoral and lumbar vertebrae. **a**–**c** μCT images and trabecular bone mineral densities (BMDs) of femurs and lumbar vertebrae in BALB/c (**a**), C57BL6 (**b**), and C3H (**c**) mice, aged 10–16 weeks, are represented in the upper and lower panels, respectively. The data shown represent the means ± SDs (*n* = 7 mice/group). *P < 0.01. Scale bar, 0.5 mm.

**Fig. 3:**
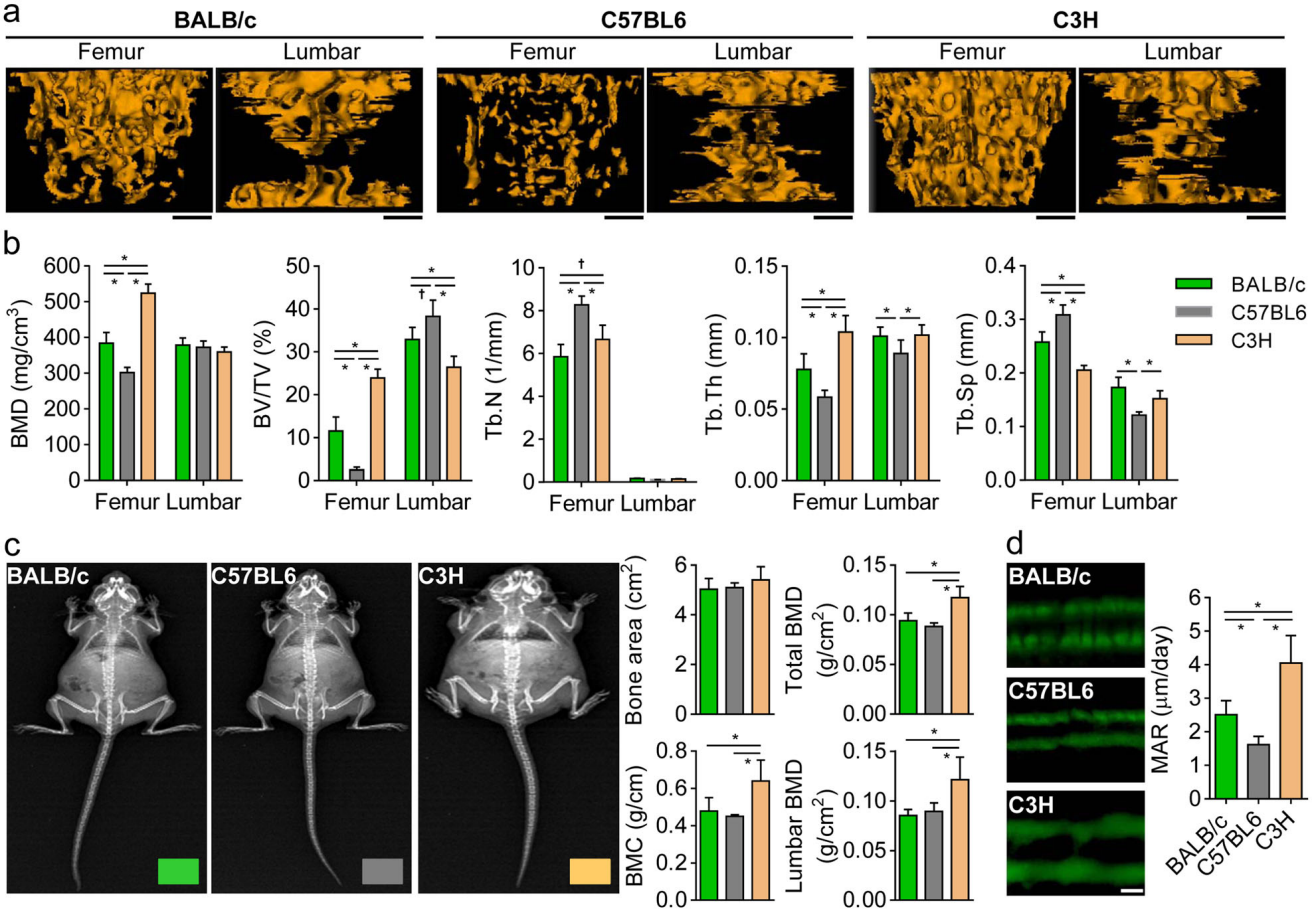
Differences in bone microstructures among the three mouse strains. Fourteen-week-old BALB/c, C57BL6, and C3H mice were subjected to detailed analysis of their bone-microarchitecture indices by performing μCT and DXA analysis. **a** μCT images of the trabecular bones of a femur and a lumbar vertebra. Scale bar, 0.5 mm. **b** Bone indices from μCT images. BMD, bone mineral density; BV/TV, bone volume/total volume; Tb.N, trabecular number; Tb.Th, trabecular thickness; Tb.Sp, trabecular separation. **c** Bone indices from DXA analysis. BMC, bone mineral content. **d** Bone-histomorphometric analysis. The mineral apposition rates (MARs) in femoral cortical bones were determined using the calcein double-labeling method. Scale bar, 10 μm. The data shown represent the means ± SDs (*n* = 7 mice/group in **b** and *n* = 10 mice/group in **c** and **d**). *P < 0.01; ^†^*P* < 0.05.

To determine whether disparity exists in bone remodeling between each mouse strain studied, we analyzed histomorphological bone parameters of femurs and lumbar vertebrae using high-resolution µCT and DXA. The µCT analysis showed that the trabecular bone densities of femurs in BALB/c mice were similar to those of lumbar vertebrae after the mice reached 14 weeks of age. Particularly, in BALB/c mice, the lumbar trabecular bone density peaked before 10 weeks of age, but the femoral trabecular bone density reached a maximum when the mice were 14 weeks old (Fig. 2a), reflecting a temporal discrepancy in the maturation stage of femoral and lumbar trabecular bones. In C57BL6 mice, the femoral trabecular bone densities were lower than those of the lumbar vertebrae (Fig. 2b). In contrast, the C3H mice showed a noticeable increase in their femoral trabecular bone densities compared to their lumbar trabecular bone densities (Fig. 2c). Whereas the lumbar trabecular bone densities were similar among all three strains, the femoral trabecular bone densities were increased in the order of C3H > BALB/c > C57BL6. The detailed bone-microarchitecture parameters (BMD, BV/TV, Tb.N, Tb.Th, and Tb.Sp) of 14-week-old mice, with a maximally saturated trabecular bone density in the femurs and lumbar vertebrae, differed among all three mouse strains (Fig. 3a, b). In addition, DXA analysis revealed higher BMC and BMD values in the whole body and lumbar vertebrae of C3H mice than in those of BALB/c and C57BL6 mice (Fig. 3c), which agreed with the μCT results. Following calcein injection, double fluorochrome-labeling measurements in the femoral diaphysis showed that the periosteal MAR increased in the following order: C3H > BALB/c > C57BL6 (Fig. 3d). This was consistent with the femoral trabecular bone density of each mouse (Fig. 2). As shown in Supplementary Fig. S2, the serum levels of osteogenic factors, including estrogen, P1NP, and osteocalcin, which participate in bone formation, were maintained at higher levels in C3H mice than in BALB/c and C57BL6 mice, showing a concurrent increase in the serum levels of RANKL (an osteoclastogenic factor) and CTX-1 (a marker of bone resorption). Together, these results indicate that the femurs and lumbar vertebrae of BALB/c, C57BL6, and C3H mice displayed different bone microarchitectures and remodeling and that C3H mice had elevated mineral apposition, bone formation, and resorption (based on P1NP and CTX-1 levels), reflecting an overall increase in bone turnover.

### Distinct estrogen deficiency-induced bone loss in the individual mouse strains

**Fig. 4:**
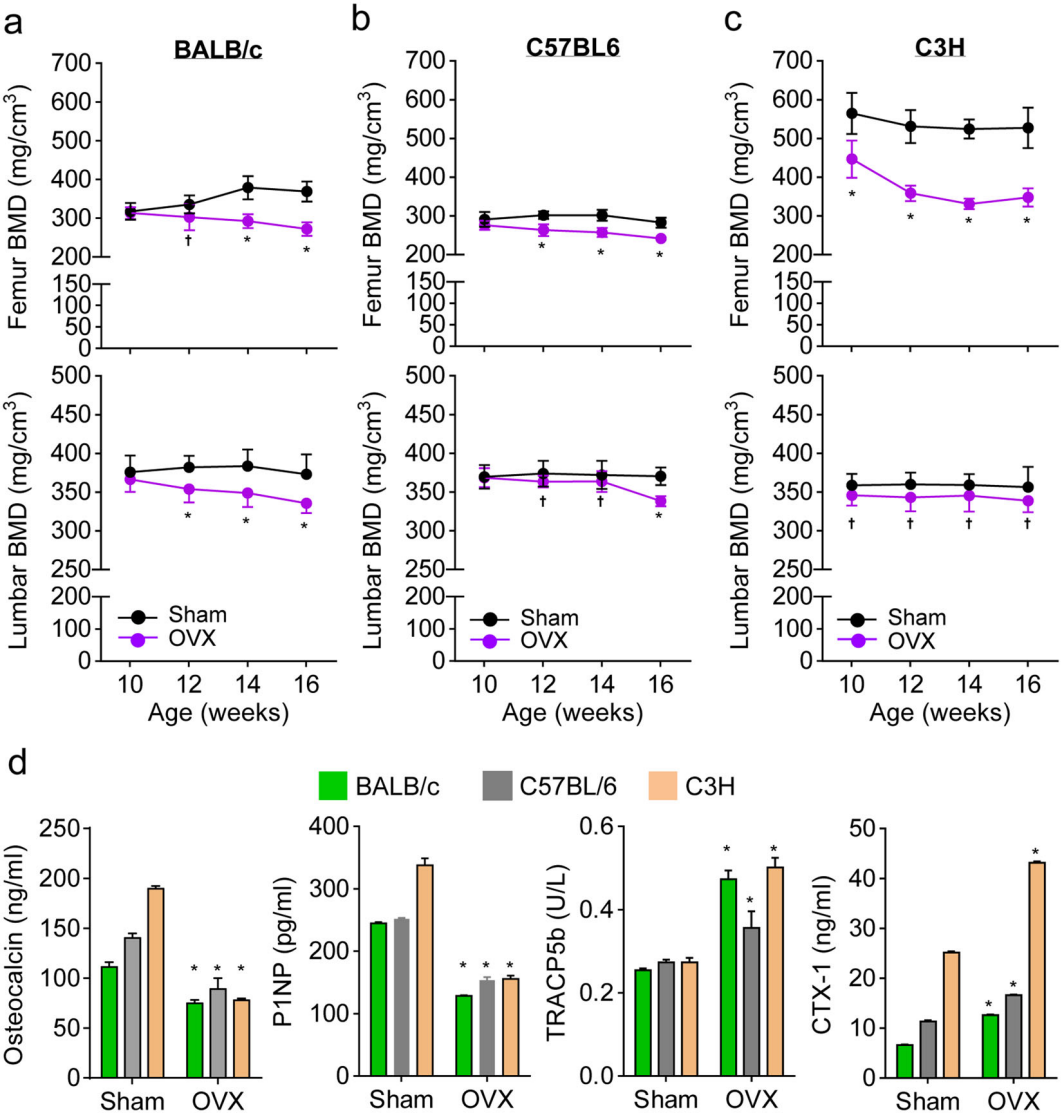
Differential bone remodeling among estrogen-deficient mouse strains. Eight-week-old mice were subjected to a sham operation or an ovariectomy (OVX). **a**, **b** After surgery, the trabecular bone mineral densities (BMDs) in the femurs and lumbar vertebrae of BALB/c (**a**), C57BL6 (**b**), and C3H (**c**) mice were analyzed between the ages of 10 and 16 weeks. D. Serum levels of bone-related parameters. Sera were obtained from mice at 6 weeks post ovariectomy, and bone-related parameters, including osteocalcin, N-terminal propeptide of type-I procollagen (P1NP), tartrate-resistant acid phosphatase 5b (TRACP5b), and type-I collagen (CTX-1), were measured using ELISA kits. The data shown represent the means ± SDs (*n* = 7 mice/group in **a**–**c** and *n* = 5 mice/group in **d**). *P < 0.01; ^†^*P* < 0.05 compared with the sham group.

Sex-steroid deficiency in males and females results in bone loss due to increased bone resorption and a relative decrease in bone formation^27^. To address whether bone remodeling among the three mouse strains was controlled by metabolic changes due to estrogen deficiency, we surgically removed the ovaries of 8-week-old female mice and monitored the mice for 8 weeks. The serum levels of 17β estradiol in BALB/c, C57BL6, and C3H mice at 8 weeks post ovariectomy decreased from 78.8 pg/ml ± 1.3 to 67.0 pg/ml ± 1.5, from 78.3 pg/ml ± 1.2 to 68.1 pg/ml ± 1.8, and from 91.9 pg/ml ± 1.8 to 61.1 pg/ml ± 1.8, respectively, showing that estrogen levels in ovariectomized mice dramatically decreased in the following order: C3H > BALB/c ≅ C57BL6 (Supplementary Fig. S3a). The body weights of 16-week-old sham-operated BALB/c, C57BL6, and C3H mice were 21.2 g ± 0.7, 24.0 g ± 1.1, and 26.7 g ± 1.7, respectively. In parallel, the body weights of ovariectomized mice tended to increase in proportion to those in the sham groups (Supplementary Fig. S3b). Based on the μCT results (Fig. 4a), the trabecular bone densities of the femurs and lumbar vertebrae of BALB/c mice gradually declined following ovariectomy; in contrast, the trabecular bone densities of ovariectomized C57BL6 mice decreased at a lower rate than those of BALB/c mice (Fig. 4b). In particular, ovariectomized C3H mice showed substantially decreased femoral trabecular bone densities compared to those of the sham groups, but the C3H mice were resistant to ovariectomy-induced trabecular bone loss in the lumbar vertebrae (Fig. 4c). When analyzing bone-related factors in the serum (Fig. 4d and Supplementary Fig. S4), we found that bone-formation marker (osteocalcin and P1NP) levels in ovariectomized mice showed a marked decrease in C3H mice compared to those in the other mouse strains, further confirming that the ovariectomized C3H mice had significantly higher levels of the bone-resorption markers TRACP5b and CTX-1. Collectively, these results indicate that metabolic changes caused by estrogen deficiency differed between the mouse strains and led to differential bone turnover in their femurs and lumbar vertebrae.

### Risedronate led to abnormal cortical bone remodeling in femurs but not in lumbar cortical bones

**Fig. 5:**
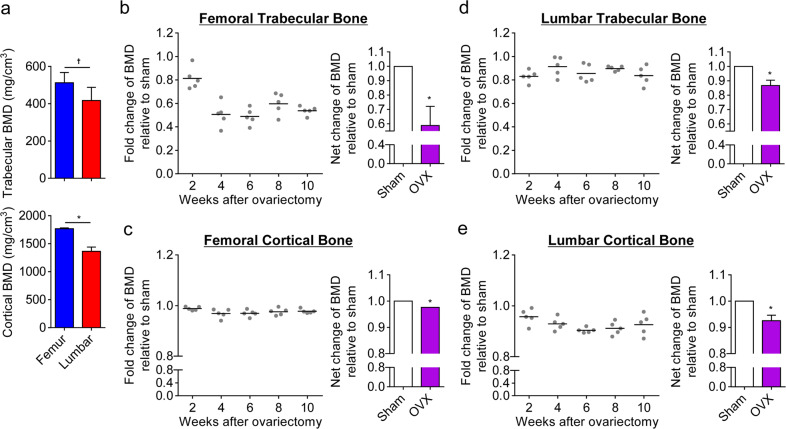
Differential trabecular and cortical bone remodeling between femurs and lumbar vertebrae in estrogen-deficient C3H mice. Female C3H mice were ovariectomized (OVX) at 8 weeks of age, and the trabecular and cortical bone densities in femurs and lumbar vertebrae were measured at 2-week intervals for 10 weeks. **a** Trabecular and cortical bone densities in the femurs and lumbar vertebrae of sham-operated mice were determined at 10 weeks after the operation. **b**–**e** Trabecular and cortical bone densities of femurs and lumbar vertebrae in ovariectomized mice were compared to those in sham-operated mice every 2 weeks for 10 weeks after surgery. The left panel indicates relative fold changes in terms of trabecular and cortical bone loss of the femurs and lumbar vertebrae in ovariectomized mice compared to the sham groups. The mean net changes in bone densities between sham-operated and ovariectomized mice are represented in the right panel. The data shown are expressed as the means ± SDs (*n* = 5 mice/group). *P < 0.01; ^†^*P* < 0.05 (compared with the sham group in **b**–**e**).

We observed that BALB/c, C57BL6, and C3H mice displayed differences in their metabolic indices, including physical activity, respiration rate, energy expenditure, and body-weight gains caused by aging and estrogen deficiency, as well as in femoral and lumbar bone remodeling in the context of normal and low estrogen levels. In contrast to BALB/c and C57BL6 mice, C3H mice had higher femoral trabecular bone densities, and estrogen deficiency rendered them more susceptible to femoral trabecular bone loss than lumbar trabecular bone loss. To more deeply analyze trabecular and cortical bone remodeling in femurs and lumbar vertebrae and to observe bone remodeling in response to bisphosphonate risedronate treatment, we selected C3H mice, which have a higher density in the femoral bones than in the lumbar vertebrae (similar to that seen in humans). The trabecular and cortical bone densities of the femurs of C3H female mice were significantly higher than those of the lumbar vertebrae (Fig. 5a). Next, we analyzed bone loss at 2-week intervals for 10 weeks in mice following ovariectomy. The femoral trabecular bone density sharply decreased at 4 weeks post ovariectomy, whereas the femoral cortical bone density showed a uniform and slight reduction following ovariectomy, revealing differences in the rates of decreasing trabecular and cortical bone densities in femurs (Fig. 5b, c). The overall loss of lumbar trabecular bone mass due to menopause was similar to that of lumbar cortical bones (Fig. 5d, e). Taken together, these results show that cancellous bone masses in femurs were reduced to a greater extent than those in lumbar vertebrae after ovariectomy, whereas the cortical bones of femurs decreased to a lesser extent than those in the lumbar vertebrae.

**Fig. 6:**
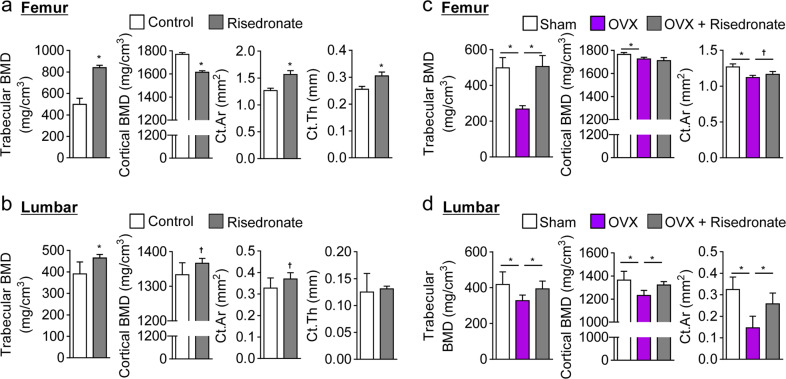
Risedronate induced abnormal femoral cortical bone remodeling in C3H mice. **a**, **b** Eight-week-old female wild-type C3H mice were subcutaneously administered risedronate (20 μg/kg) every 2 weeks for 10 weeks. Trabecular and cortical bone morphometries were analyzed in the femurs (**a**) and lumbar vertebrae (**b**). **c**, **d** After 8-week-old C3H mice were subjected to a sham operation or ovariectomy (OVX), they were administered risedronate (20 μg/kg) as performed in the experiments represented in **a** and **b**, after which trabecular and cortical bone morphometries were analyzed in their femurs (**c**) and lumbar vertebrae (**d**). BMD, bone mineral density; Ct.Ar, cortical area; Ct.Th, cortical thickness. The data shown represent the means ± SDs (*n* = 5 mice/group). *P < 0.01; ^†^*P* < 0.05 (compared with control in **a** and **b**).

Based on the differences observed in trabecular and cortical bone remodeling of the femurs and lumbar vertebrae, we finally tested the responsiveness of bone remodeling to risedronate treatment under the conditions of normal and low estrogen levels in C3H female mice. To do this, we subcutaneously injected risedronate (a potent blocker of osteoclastic bone resorption)^28^ into 8-week-old mice with normal estrogen levels at 2-week intervals for 10 weeks. Risedronate treatment led to increased serum levels of bone-formation markers (e.g., osteocalcin, PTH, and vitamin D; Supplementary Fig. S5) and a moderate increase in only the trabecular bone density of femurs and in both the trabecular and cortical bone densities of lumbar vertebrae (Fig. 6a, b). In contrast to the positive effect of risedronate at most sites of bone remodeling, risedronate treatment reduced the density and enlarged the area and thickness of femoral cortical bones (Fig. 6a), manifesting in abnormal femoral cortical bone remodeling. Strikingly, this negative effect was not observed in estrogen-deficient osteoporotic mice, revealing that bone density in estrogen-deficient mice was comparable to that in sham-operated mice (Fig. 6c, d). These results suggest that risedronate treatment leads to defective cortical bone remodeling in femurs with normal bone density but that the defect does not occur in osteoporotic disease. Consequently, our findings suggest that risedronate induces an abnormal microarchitecture in normal femoral cortical bone, resulting in a high frequency of fragility fractures, such as AFFs.

## Discussion

The cumulative results of previous results have provided strong support for an interplay between metabolic activity and bone remodeling^29,30^. In the central axis of their mutual connection, estrogen deficiency in postmenopausal women leads to metabolic defects with increased body fat mass and weight, which is accompanied by bone loss caused by excessive bone resorption and/or impaired bone formation. Estrogen regulates lipid metabolism with increased high-density lipoprotein and decreased low-density lipoprotein and suppresses binge eating more prevalently in women than in men^31^. Estrogen can also induce bone formation and inhibit bone resorption, functioning in anabolic and anti-catabolic bone metabolism^32^. With regard to the tuning of bone remodeling by metabolic regulators, adipokines (for instance, leptin and adiponectin) expressed and secreted from adipocytes can act as messengers between adipose tissue and bones, which shows that these factors regulate both energy homeostasis and bone remodeling by arbitrating between bone resorption and formation^30,33,34^. Inversely, osteocalcin, which is the most abundant, bone-specific noncollagenous protein secreted from osteoblasts and present in bone matrix and blood, can help regulate glucose homeostasis, thereby contributing to insulin synthesis and peripheral insulin sensitivity^35^. Consistent with the interplay between metabolic activity and bone remodeling, we observed here that differences in metabolic activities among BALB/c, C57BL6, and C3H mice may help determine the bone density and bone turnover rate. In this study, we found that C3H mice had the highest values for fat and lean body mass, physical activity in the active dark phase, RQ (determined as the VCO_2_/VO_2_ ratio) and EE compared to sex- and age-matched BALB/c and C57BL6 mice, and displayed a concurrent increase in the BMD, MAR, and bone turnover rate (based on serum P1NP and CTX-1 levels). These findings suggest that the degree of active and dynamic processes involved in bone remodeling increases in proportion to metabolic activity.

Genetic factors have been reported to modulate various aspects of bone physiology, including the peak bone mass, bone structure, and bone composition^36^. Animal models have been established as valuable tools for experimentally defining the genetic regulation of bone mass and identifying the genetic determinants of bone structure that have been validated in humans^37,38^. In particular, inbred mice that have a homogeneous genetic background are useful models for studying genetic effects on bone structure and for examining variations in bone phenotypes under strictly controlled environmental conditions^37^. In addition, skeletal responses to ovariectomy were reported to vary in site- and compartment-specific manners among inbred strains of mice, indicating that the genetic regulation of bone loss was induced by estrogen deficiency^37^. Here we used three inbred mouse strains (BALB/c, C57BL6, and C3H) to study differential bone remodeling of femurs and lumbar vertebrae in healthy and estrogen-deficient mice. Among the mouse strains studied, C3H mice had the highest femoral trabecular BMD, BV, and thickness; these parameters were lowest in C57BL6 mice displaying a higher femoral Tb.N and separation. In contrast, lumbar trabecular bone density was similar among all three mouse strains. Consequently, C3H mice had higher trabecular bone densities in the femurs than in the lumbar vertebrae, in contrast to the other strains. This bone phenotype of C3H mice is similar to that of humans. In bone remodeling caused by metabolic alterations, C3H mice showed dramatically decreased serum estrogen levels and a rapid increase in body weight after ovariectomy compared to the other strains. More specifically, the ovariectomized C3H mice displayed significantly decreased bone-formation markers (osteocalcin and P1NP) and significantly increased bone-resorption markers (TRACP5b and CTX-1), showing that C3H mice had the greatest ovariectomy-induced femoral trabecular bone losses but were resistant to lumbar trabecular bone loss induced by ovariectomy. These results suggest that metabolic alterations arise due to the genetic background, leading to differential pathogenesis and regulation of trabecular and cortical bone remodeling in femurs and lumbar vertebrae.

Bisphosphonates comprise a class of drugs that inhibit osteoclast-mediated bone loss due to osteoporosis, multiple myeloma, Paget’s disease, and hypercalcemia in cancer and bony metastasis, and they reduce the risk of bone fractures in postmenopausal women by up to 50%^39^. Side effects of long-term bisphosphonate treatment have been associated with AFFs^40^. The femoral cortical bones of bisphosphonate-treated patients with atypical fractures are harder and more mineralized than those with typical fractures. Interestingly, in this study, we observed that femoral cortical bones from healthy mice treated with bisphosphonate risedronate were enlarged and had a low density, but this side effect was not observed in mice with ovariectomy-induced femoral cortical bone loss. Our results showed that the adverse effect of treatment with the anti-osteoporotic drug risedronate on femoral cortical bone remodeling could be explained as follows. The structural stiffness depends on both the intrinsic material properties and the shape and size of an object. The bending stiffness of a hollow cylinder can be calculated as the product of its *E*-modulus and the second moment of inertia, *I* = *π* × (outer diameter^4^ − inner diameter^4^)/64. Therefore, the strength of a hollow structure is generally stronger than that of a solid structure. During physiological bone remodeling, the inner cavity of a femur, one of the long and tubular bones of the arms and legs, becomes enlarged by resorbing the bone, which increases the size of the hollow interior of the femur. With a structure that is similar to that of a hollow cylinder, the femur (with its expanded hollow center) provides maximal strength with a minimal amount of bone constituents, enabling resistance to internal and external stresses in the form of bending forces. Ordinarily, femoral bones are continuously renewed to increase the shaft size and decrease the cortical bone thickness from birth into adulthood. Based on the principle of the second moment of inertia, tubular bones with increased outer and inner diameters in adulthood have a higher bending stiffness than those at a younger age. Consequently, as an individual grows older, the outer and inner femoral diameters are increased by growing bone and resorbing cortical bone, respectively, which helps reduce fractures of the osteoporotic diaphyseal femurs in older adults^41–43^. In contrast, the results of this study showed that risedronate led to a narrowing of the inner diameter of the femur, caused by growing cortical bones of a limited size. Thus, we suggest that abnormal femoral cortical bones caused by risedronate treatment can lead to femur fragility.

In summary, we found that risedronate treatment in mice with normal femoral remodeling led to abnormal femoral cortical bones with an enlarged thickness and low bone density, which can promote AFFs. This negative effect was not found in mice with ovariectomy-induced femoral cortical bone loss. Together, our findings suggest that AFFs caused by long-term bisphosphonate treatment should be considered when bisphosphonate-related agents are prescribed to treat patients with discordances between the femurs and lumbar vertebrae, particularly in the case of osteoporotic lumbar vertebrae and a normal femoral bone density.

## Supplementary information

Supplementary Figures
