# Supplementary material for: Metabolic activities affect femur and lumbar vertebrae remodeling, and anti-resorptive risedronate disturbs femoral cortical bone remodeling
Source: Exp Mol Med. 2021 Jan 12;53(1):103–14. doi: 10.1038/s12276-020-00548-w (PMC8080628; doi:10.1038/s12276-020-00548-w)
Supplement: Supplementary file 1 — Supplementary Figures [file 12276_2020_548_MOESM1_ESM.docx]

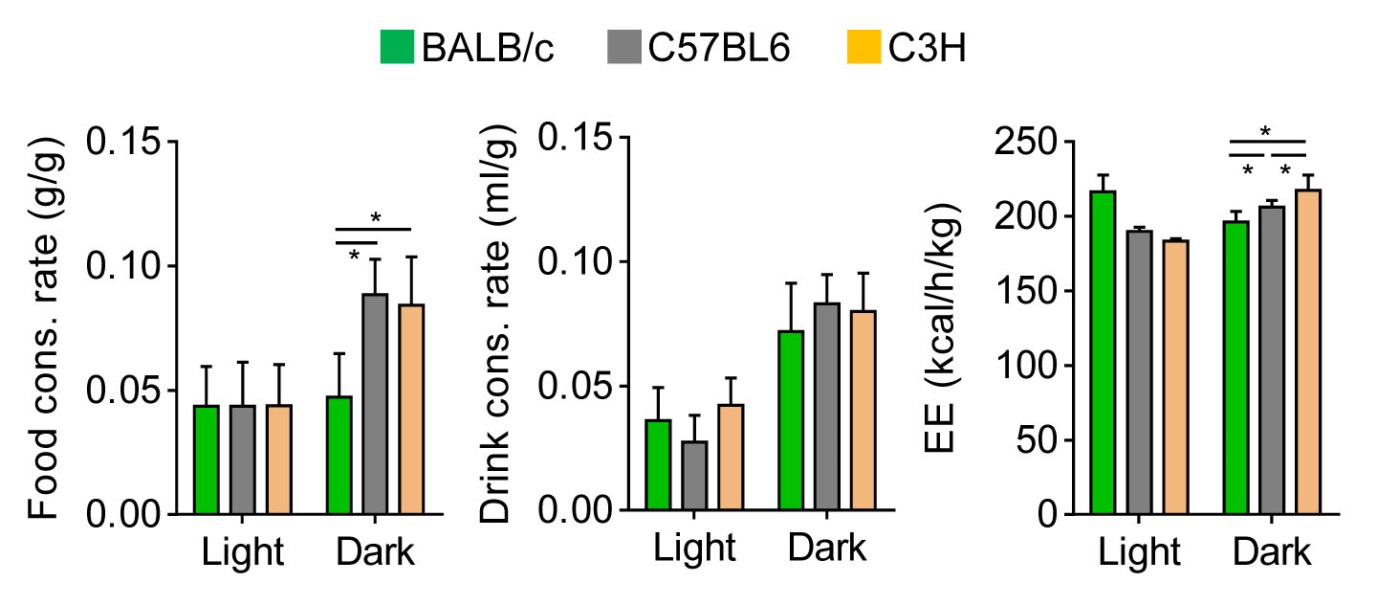


**Fig. S1 Metabolic-parameter analysis using a metabolic cage.** Fourteen-week-old female mice were maintained in metabolic cages, controlled by a 12 h: 12 h light/dark cycle for 48 h. Food consumption, drink consumption, and energy expenditure (EE) were calculated by monitoring cumulative values for the mice in the metabolic cages. Food-consumption rates, drink-consumption rates, and EEs were normalized to body weights. The data shown represent the means ± SDs (n = 10 mice/group). ^*^*P* < 0.01.


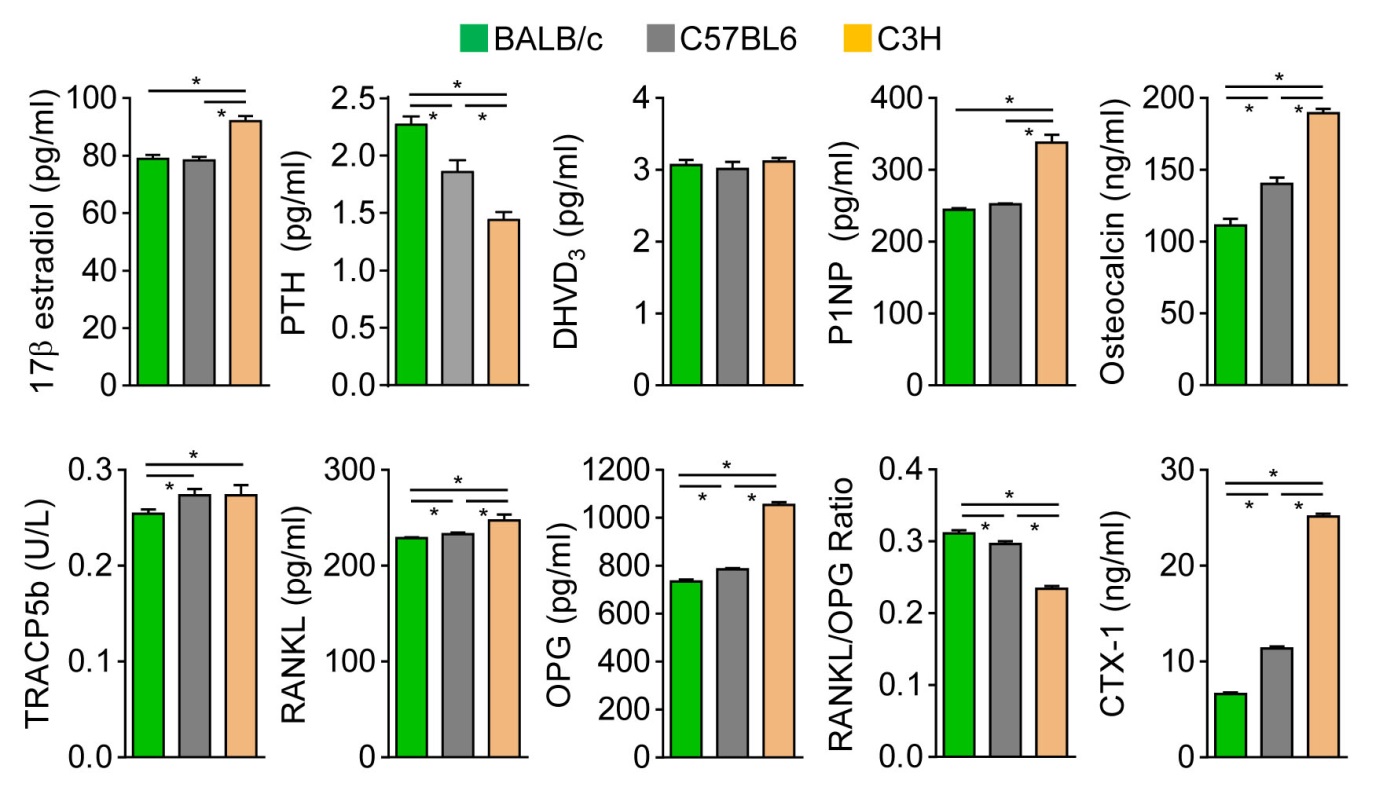


**Fig. S2 Analysis of serum bone-related parameters.** Bone-related parameters including bone-formation markers [17β estradiol, PTH, 1,25-dihydroxyvitamin D_3_ (DHVD_3_), P1NP, and osteocalcin] and bone-resorption makers (TRACP5b, RANKL, OPG, and CTX-1) were determined in sera from 14-week-old female BALB/c, C57BL6, and C3H mice, using ELISA kits. The data shown represent the means ± SDs (n = 5 mice/group). ^*^*P* < 0.01.


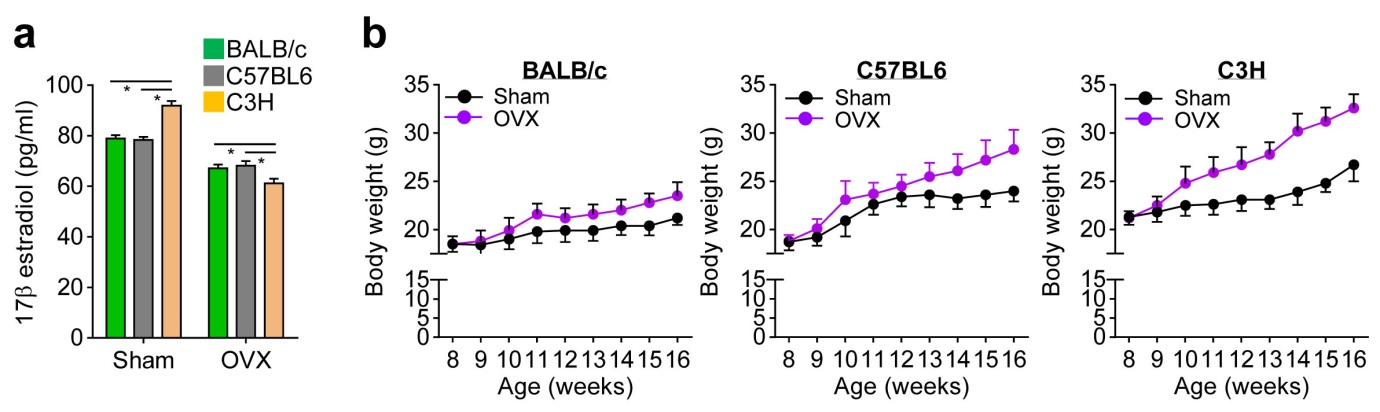


**Fig. S3 Body-weight changes in ovariectomized mice.** Eight-week-old female mice were subjected to a sham operation or an ovariectomy (OVX) and monitored for 8 weeks. **a**. Serum estrogen (17β estradiol) concentrations in mice were measured using an ELISA kit at 6 weeks post-ovariectomy. **b**. Body weights of mice were weighed using an analytical balance every week for 8 weeks post-ovariectomy. The data shown represent the means ± SDs (n = 5 mice/group in A and n = 7 mice/group in B). ^*^*P* < 0.01.


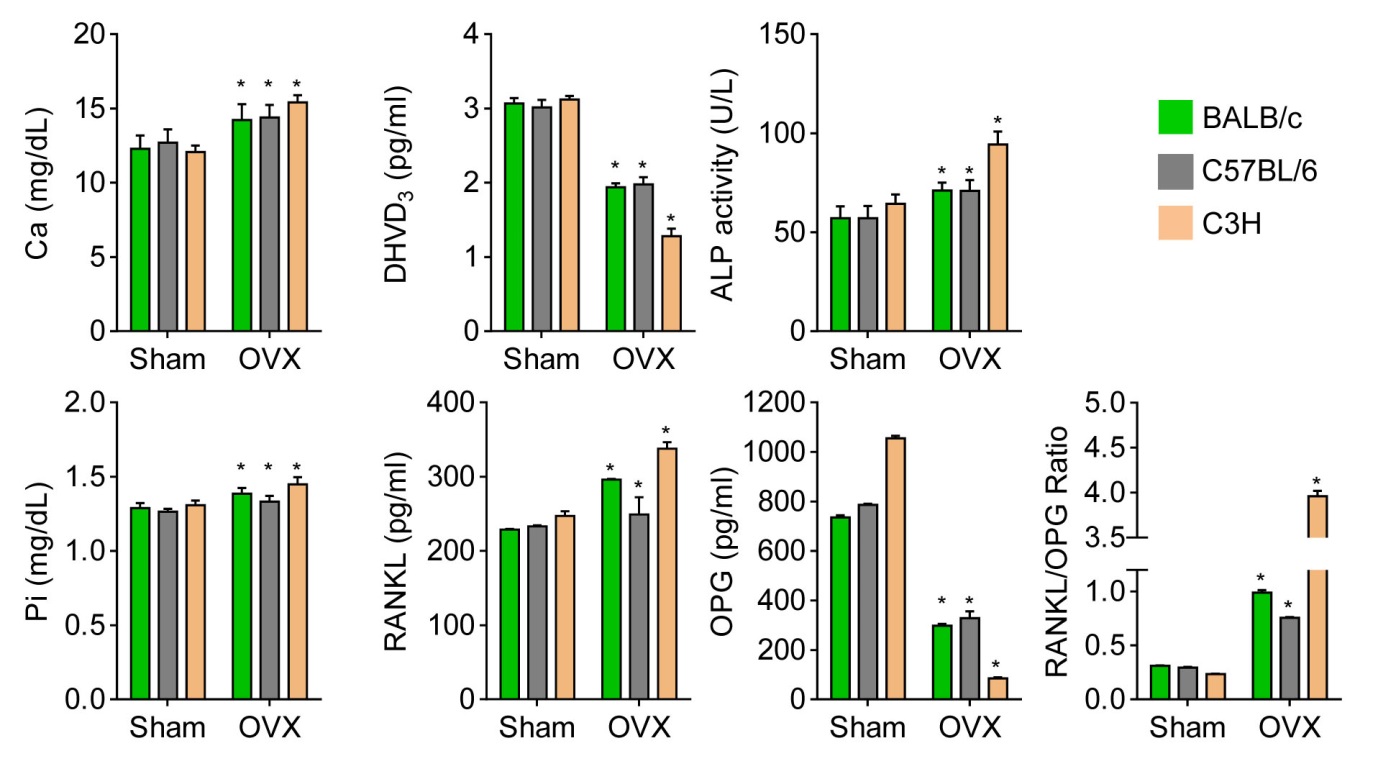


**Fig. S4 Changes in serum bone-related parameters in ovariectomized mice.** After a sham operation or an ovariectomy (OVX) in 8-week-old female mice, sera were obtained from mice at 6 weeks post-ovariectomy and bone-related factors including bone-formation factors (vitamin D; DHVD_3_, and alkaline phosphatase [ALP]), and bone-resorption factors (RANKL and OPG) were analyzed using ELISA kits. Bone constituents (calcium and phosphate) were determined using appropriate assay kits. The data shown represent the means ± SDs (n = 5 mice/group). ^*^*P* < 0.01 compared with sham group.


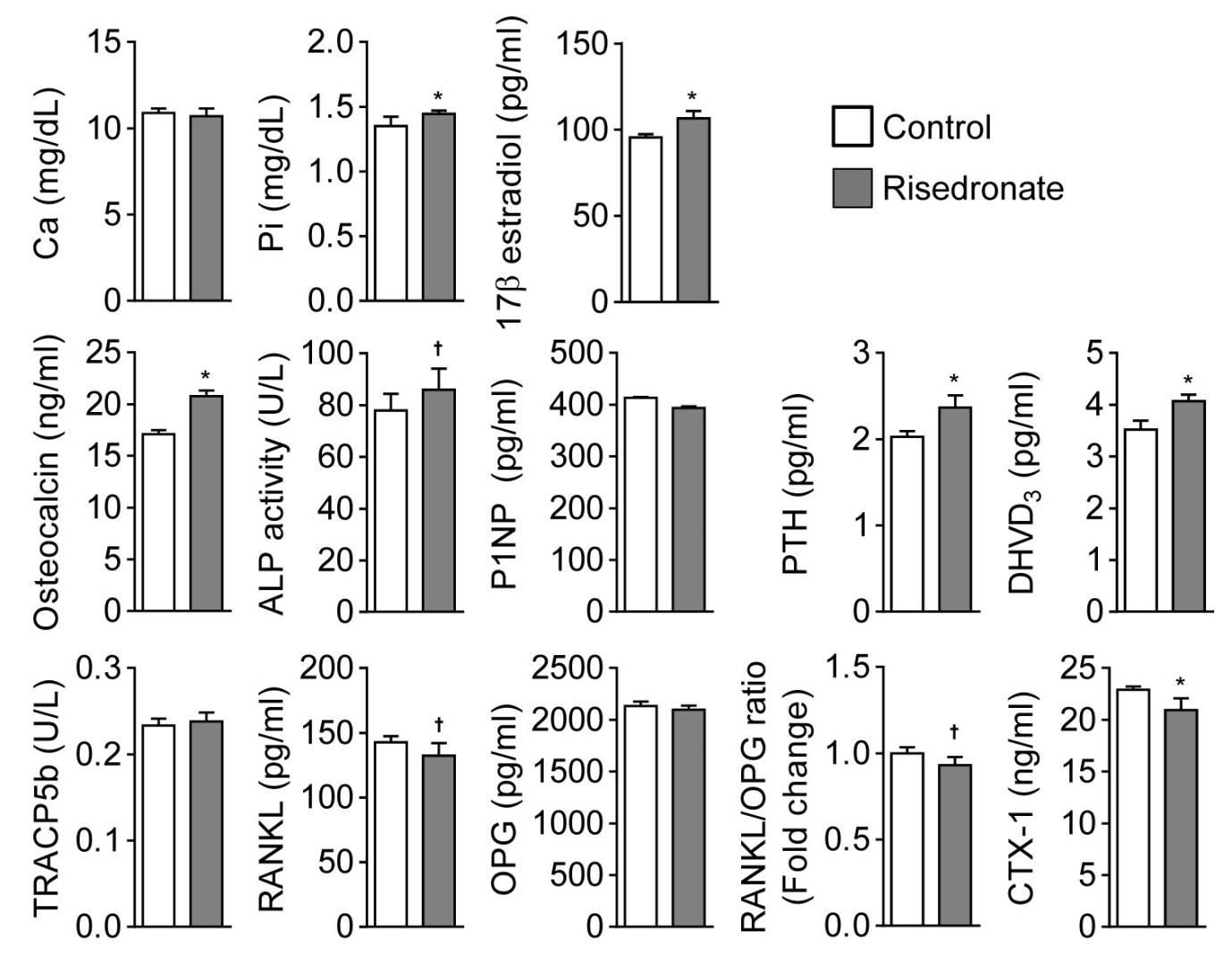


**Fig. S5 Changes in serum bone-related parameters of C3H mice treated with risedronate.** Eight-week-old C3H mice were subcutaneously injected with risedronate (20 μg/kg) every 2 weeks for 10 weeks. Bone-related factors were analyzed in sera from control and risedronate-treated mice using ELISA kits (for bone-formation markers: 17β estradiol, osteocalcin, ALP, P1NP, PTH, and DHVD3; for bone-resorption markers, TRACP5b, RANKL, OPG, and CTX-1) or assay kits (for calcium and phosphate). The data shown represent the means ± SDs (n = 5 mice/group). ^*^*P* < 0.01; ^†^*P* < 0.05 (compared with control).
